# Zero echo time MRI improved detection of erosions and sclerosis in the sacroiliac joint in comparison with LAVA-flex

**DOI:** 10.3389/fendo.2023.1167334

**Published:** 2023-05-29

**Authors:** Churong Lin, Dong Liu, Huiquan Wen, Budian Liu, Liudan Tu, Jieruo Gu

**Affiliations:** ^1^ Department of Radiology, The Third Affiliated Hospital of Sun Yat-Sen University, Guangzhou, Guangdong, China; ^2^ Department of Rheumatology, The Third Affiliated Hospital of Sun Yat-Sen University, Guangzhou, Guangdong, China

**Keywords:** zero echo time (ZTE), MRI, LAVA, axSpA, structural lesions

## Abstract

**Background:**

T1-weighted spoiled 3D Gradient Recalled Echo pulse sequences, exemplified by Liver Acquisition with Volume Acceleration-flexible MRI (LAVA-Flex), are currently the preferred MR sequence for detecting erosions of the sacroiliac joint (SIJ). However, zero echo time MRI (ZTE) is recently reported to provide excellent visualization of the cortical bone.

**Purpose:**

To directly compare the diagnostic accuracy of ZTE and LAVA-Flex in the detection of structural lesions of the SIJ, including erosions, sclerosis and joint space changes.

**Materials and methods:**

Two readers independently reviewed the ldCT, ZTE and LAVA-Flex images of 53 patients diagnosed as axSpA and scored the erosions, sclerosis and joint space changes. Sensitivity, specificity and Cohen’s kappa (κ) of ZTE and LAVA-Flex were calculated, while McNemar’s test was employed to compare the two sequences for the positivity of detecting the structural lesions.

**Results:**

Analysis of diagnostic accuracy showed a higher sensitivity of ZTE in comparison with LAVA-Flex in the depiction of erosions (92.5% vs 81.5%, p<0.001), especially first-degree erosions (p<0.001) and second-degree erosions (p<0.001), as well as sclerosis (90.6% vs 71.2%, p<0.001), but not joint space changes (95.2% vs 93.8%, p=0.332). Agreement with ldCT was also higher in ZTE in the detection of erosions than LAVA-Flex as indicated by the κ values (0.73 vs 0.47), as well as in the detection of sclerosis (0.92 vs 0.22).

**Conclusion:**

With ldCT as the reference standard, ZTE could improve diagnostic accuracy of erosions and sclerosis of the SIJ in patients suspected of axSpA, in comparison with LAVA-Flex.

## Introduction

Axial spondyloarthritis (axSpA) belongs to the group of diseases known as inflammatory arthropathies, featuring inflammation in the axial skeleton (1). Since the introduction of the Assessment of SpondyloArthritis International Society (ASAS) classification criteria of axSpA, MRI has been increasingly employed to visualize inflammation in the sacroiliac joints (SIJ), and its role in the diagnosis of axSpA has been thereby cemented (2, 3). Typical lesions seen in the SIJ MRI in patients with axSpA include inflammatory lesions such as bone marrow edema (BME) and structural lesions such as bone erosions and ankylosis (4, 5).

Conventional MRI sequences such as T1-weighted (T1w) sequence have limited value in the imaging of cortical bone, since the cortical bone has relatively low proton density and presents rapid transverse relaxation of the tissue (6). Currently, volumetric interpolated breath-hold examination (VIBE), is considered the preferred MRI technique for detecting erosions in the SIJs with higher sensitivity and superior reliability compared to the T1-weighted sequence (7, 8). Liver Acquisition with Volume Acceleration MRI (LAVA, GE Healthcare) is a similar sequence to VIBE (Siemens Healthcare), both of which are T1w spoiled 3D Gradient Recalled Echo pulse sequences (T1w 3D-GRE) from different vendors (9). LAVA-Flex can generate water-only, fat-only, in-phase and out-of-phase images from a single acquisition. The water-only images could be seen as the counterpart of VIBE.

However, a number of novel MRI sequences with CT-like bone contrast have emerged ever since. Zero echo time MRI (ZTE) is an exemplary technique for the visualization of osseous structures since it employs zero echo times, enabling the precise depiction of cortical bone contours (9). Previous studies investigating ZTE in the imaging of bone structures in the head, shoulder and vertebra concluded that its contrast almost paralleled CT (10–12).

The objective of this study is to investigate the diagnostic accuracy of ZTE in the detection of structural lesions, including erosions, sclerosis and joint space changes, in the SIJs in patients with axSpA, in comparison with LAVA-Flex using low-dose CT (ldCT) as the reference standard. It is hypothesized that ZTE possessed higher sensitivity and better inter-reader agreement in the detection of bone erosions than T1w 3D-GRE images.

## Materials and methods

### Design

This study was a cross-sectional diagnostic study investigating ZTE for the detection of structural lesions in comparison with LAVA-Flex in patients with axSpA. This study was approved by the Ethical Committee of the hospital. Written informed consent was obtained from patients prior to the inclusion of this study ([2020]02-031-01).

### Study population

All patients diagnosed as axSpA were considered eligible for this study and were consecutively recruited at the outpatient clinic from September 1^st^, 2021 to January 31^st^, 2022. Inclusion criteria were diagnosis of axSpA by an expert rheumatologist and fulfillment of ASAS classification criteria for axSpA (2). Exclusion criteria consisted of age below 18 years, concomitant malignancies, pregnancy and contraindications for MRI or CT. Both MRI scans and ldCT scans were obtained on the same day of the visit.

### MRI protocol

All patients underwent MRI scanning of the sacroiliac joints in the supine position using a 3.0 T superconducting MR scanner (SignaTM Architect, GE Healthcare, Milwaukee, WI) with an anterior 30-channel and posterior 40-channel adaptive image receive (AIR) radiofrequency coil. The routine SIJ MRI examination consisted of T2-weighted fat-suppressed turbo spin echo (T2-FS) sequence, T1-weighted images (T1WI) sequence, T1-weighted images with fat saturation (T1-FS) sequence in a semi-coronal orientation and T2-FS sequences in a semi-axial orientation for the SIJ were available. In addition, a ZTE sequence and a LAVA-Flex sequence were performed. Scan parameters of each MRI sequence could be seen in **Table 1**.

**Table 1:** Imaging parameters of MRI sequences.

Parameters	ZTE	LAVA-Flex	T1WI	T1-FS	T2-FS	T2-FS
Plain	Coronal	Coronal	Coronal	Coronal	Coronal	Transverse
No.of section	60	52	18	18	18	18
Section thickness (mm)	1.4	1.4	3	3	3	3
Section gap (mm)	0	0	0.6	0.6	0.6	0.6
Field of view (mm)	280*280	280*280	240*240	240*240	240*240	280*280
Voxel size (mm)	0.9*0.9*1.1	1.2*1.2*1.4	0.8*0.9*3	0.9*0.9*3	0.8*0.9*3	0.8*1.0*3
Repetition time (msec)	831	7.1	624	725	4304	4561
Echo time (msec)	0	1.9	7	7	68	68
Acquisition time	3:04	1:30	1:30	1:54	2:05	1:36

ZTE, zero echo time; LAVA-Flex, liver acquisition volume acceleration -flexible; T2-FS, T2-weighted fat-suppressed turbo spin echo sequence; T1WI, T1-weighted images; T1-FS, T1-weighted images with fat saturation.

### ldCT protocol

ldCT was employed as the standard of reference for the detection of structural lesions. All CT examinations were performed on a third-generation dual-source CT (SOMATOM Force; Siemens Healthineers, Germany). The number of acquired slices was 384 (2×192) slices. Tube voltage was set to Sn100 kV, where Sn indicates the use of a 0.64 mm tin filter, which modifies the spectrum so that exposure to radiation is reduced by blocking low-energy photons (13). The ldCT scanning parameters for the Sn100-kV protocol were as follows: automated tube current modulation (Care Dose 4D) with reference value 600 mAs, rotation time 0.5s, pitch 0.55, 192×0.6 mm detector collimation, and reconstruction slice thickness 1.4 mm; The volume CT dose index (CTDI_vol_) and dose-length product (DLP) were registered from the study protocol for every patient. The mean radiation exposure of ldCT was calculated to be 0.50 mSv (SD 0.07) with a maximum of 0.59 mSv.

### Image evaluation

The MRI and CT scans were evaluated independently by one radiologist with 9 years’ experience in musculoskeletal imaging evaluation and one rheumatologist with 2 years’ experience in musculoskeletal imaging evaluation. Both observers were blinded to the clinical information and other sequences, with no prior knowledge of the patients’ information. MRI and CT scans were all anonymized and presented to the observers in a random order. Training sessions were assigned to the two observers to score 10 test images in order to reach consensus.

**Figure 1:**
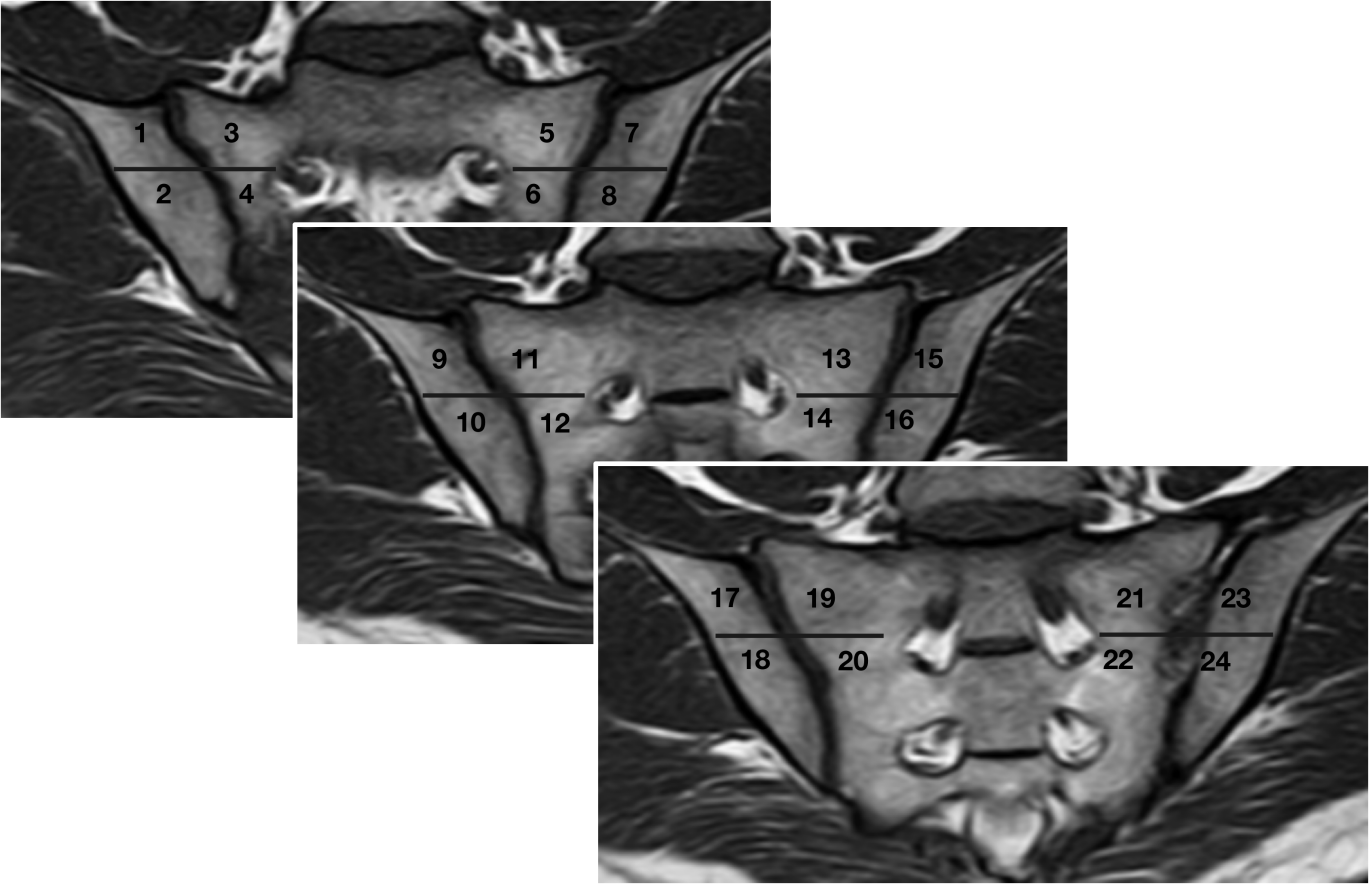
Slice selection for image evaluation and localization of all 24 regions. Anterior slice: ventral to the slices where the sacral neuroforamina was seen. Middle slice: the anterior sacral neuroforamina and the sacral bone could be seen. Posterior slice: the entheseal joint compartment stretched to the posterior inferior part of the joint. Oblique coronal slice orientation is a prerequisite for the scoring system.

Structural lesions, including erosions, sclerosis and joint space changes, were scored based on a method described by Diekhoff et al (14). with modifications to the scoring of sclerosis. The scoring method is summarized in **Table 2**. Three slices were selected for interpretation. On each slice, every SIJ was divided into 4 regions, with a sum of 24 regions per patient. The anterior slice was defined as ventral to the slices where the sacral neuroforamina was seen. The middle slice was located by the visualization of the anterior sacral neuroforamina and the sacral bone. The posterior slice was defined where the entheseal joint compartment stretched to the posterior inferior part of the joint. The selection of the three slices was exemplified in **Figure 1**. The observers were also asked to record their confidence of identifying structural lesions on a 0-to-10 scale for each slice.

**Table 2:** Scoring system for the structural lesions of the sacroiliac joint.

Erosions ^a^		Sclerosis ^a^		Joint space changes ^b^	
0-	No erosions	0-	No sclerosis or limited sclerosis (less than 10 mm)	0-	No joint space changes
1-	Small isolated erosions (1–2) or questionable single erosion	1-	Evident sclerosis (≥10 mm)	1-	Questionable widening or narrowing
2-	Definite erosions (3–5; <3 mm) or larger single erosion (>3 mm)			2-	Pseudowidening
3-	Multiple (>5) or confluent erosions			3-	Partial ankylosis
				4-	Extensive/total ankylosis

a Erosions and sclerosis were scored on the level of quadrant of each sacroiliac joint.

b Joint space changes were scored on the joint level on each slice.

### Statistical analysis

Statistical analysis was done using R version 3.3. Characteristics of study subjects were summarized with standard descriptive statistics. With ldCT as the standard of reference, sensitivity, specificity, positive predictive value (PPV), negative predictive value (NPV) and accuracy of ZTE and LAVA-Flex were calculated. By using ldCT as the reference standard, the agreement of ZTE and LAVA-Flex with ldCT was evaluated with unweighted Cohen’s Kappa (κ value). Such parameters were calculated on the level of quadrants for erosions and sclerosis, while for joint space changes these parameters were calculated on the joint level. A contingency table analysis was conducted comparing ZTE and LAVA-Flex in the detection of structural lesions. The diagnostic performance of the two MRI sequences was compared using McNemar’s test.

The sum scores of different structural lesions and diagnostic confidence of ldCT, ZTE and LAVA-Flex were tested for significant differences by Kruskal Wallis’ test. The inter-observer reliability for different structural lesions was assessed with κ value.

A p-value less than 0.05 is considered statistically significant.

## Results

### Demographic characteristic

A total of 53 axSpA patients were included in this study. Twenty-three patients were classified as non-radiographic axSpA (mean age, 33.5 ± 8.29), and 30 patients were classified as radiographic axSpA (mean age, 31 ± 5.88). Of the 53 included patients, 34/53 (64.15%) were male and 19/53 (35.85%) were female. The demographic characteristics of the included patients could be seen in **Table 3**.

**Table 3:** Demographic characteristics of the patient population.

	nr-axSpA (n=23)	r-axSpA (n=30)	P value
Age (y)	33.5 ± 8.29 (18-49)	31 ± 5.88 (22-45)	0.209
Male patient	12 (52.17%)*	22 (73.33%)*	0.193
Disease duration (y)	6.11 ± 4.91 (0.2-20)	6.97 ± 5.56 (0.1-20)	0.633
BMI (kg/m2)	22.8 ± 3.51 (18-30)	22.3 ± 4.22 (16.7-35.3)	0.830
Smoking history	4 (17.39%)*	11 (36.67%)*	0.216
HLA-B27 positive	19 (82.61%)*	28 (93.33%)*	0.433
ASDAS-CRP	1.69 ± 1.01 (0.31-4.07)	2.14 ± 0.97 (0.61-3.81)	0.102
BASDAI	3.04 ± 3.55 (0-8.64)	2.25 ± 2.84 (0-5.02)	0.753
bDMARDs	9 (39.13%)*	13 (43.33%)*	0.979

Unless otherwise specified, data are means ± standard deviation, with ranges in parentheses. BMI, body mass index; ASDAS, Ankylosing Spondylitis Disease Activity Score, BASDAI, Bath Ankylosing Spondylitis Disease Activity Index; bDMARDs, biologic disease-modifying antirheumatic drugs.

* Data are numerators and denominators, with percentages in parentheses.

### Diagnostic accuracy of erosions

**Figure 2:**
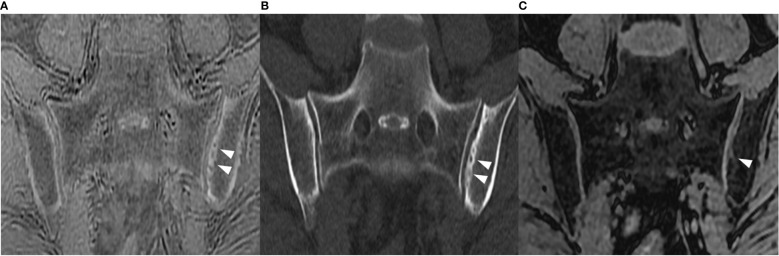
Semi-coronal images in a 30-year-old woman with sacroiliitis. **(A)** ZTE, **(B)** ldCT, and **(C)** LAVA-Flex. Obvious second-degree erosions on the left iliac surface could be clearly exhibited on ZTE and ldCT (white arrowhead) but only graded as first-degree on LAVA-Flex. ldCT, low-dose CT; ZTE, Zero echo time; LAVA-Flex, Liver Acquisition with Volume Acceleration-flexible.

**Figure 3:**
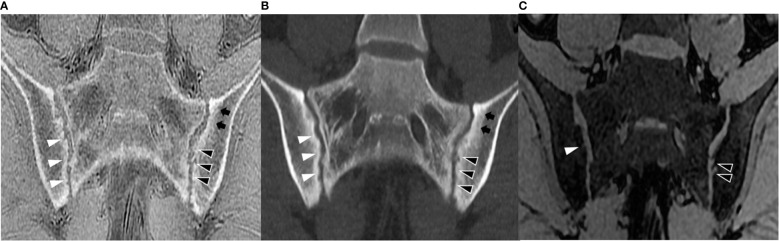
Semi-coronal images in a 45-year-old man with sacroiliitis. **(A)** ZTE, **(B)** ldCT, and **(C)** LAVA-Flex. Multiple erosions on the right sacroiliac joint (white arrowhead) and the left sacroiliac joint (black arrowhead) were graded as third-degree on ZTE and ldCT but graded as first-degree on the right and second-degree on the left on LAVA-Flex; both ZTE and ldCT could accurately exhibit sclerosis (small black arrow), but such sclerosis could be easily confused with fat deposition. ldCT, low-dose CT; ZTE, Zero echo time; LAVA-Flex, Liver Acquisition with Volume Acceleration-flexible.

The analysis of diagnostic accuracy exhibited that ZTE MRI had higher sensitivity (1582/1710 [92.5%] vs 1393/1710 [81.5%]), accuracy (2391/2544 [94.0%] vs 2202/2544 [86.6%]) and κ value (0.73 vs 0.47) in the detection of erosions on the quadrant level, in comparison with LAVA-Flex, while specificity was comparable (809/834 [97.0%] vs 809/834 [97.0%]) (**Table 4**). Contingency table analysis revealed that ZTE is superior to LAVA-Flex in the detection of erosions (p<0.001), especially the first-degree erosions (p<0.001) and second-degree erosions (p<0.001), while the positivity is not significantly different for third-degree erosions (p=0.505) (**Table 5**). Examples demonstrating improved detection of erosions by ZTE were illustrated in **Figures 2**, **3**.

**Table 4:** Diagnostic accuracy of ZTE and LAVA-Flexin the detection of structural lesions.

Parameter	Sensitivity (%)	Specificity (%)	PPV (%)	NPV (%)	Accuracy (%)	κ value (vs ldCT)
Erosion						
ZTE	1582/1710 (92.5)	809/834 (97.0)	1582/1607 (98.4)	809/937 (86.3)	2391/2544 (94.0)	0.73 (0.71-0.75)
LAVA-Flex	1393/1710 (81.5)	809/834 (97.0)	1393/1418 (98.2)	809/1126 (71.8)	2202/2544 (86.6)	0.47 (0.45-0.49)
Sclerosis						
ZTE	616/680 (90.6)	1848/1864 (99.1)	616/632 (97.5)	1848/1912 (96.7)	2464/2544 (96.9)	0.92 (0.90-0.93)
LAVA-Flex	484/680 (71.2)	1073/1864 (57.6)	484/1275 (38)	1073/1269 (84.6)	1557/2544 (61.2)	0.22 (0.19-0.26)
Joint space change						
ZTE	340/357 (95.2)	251/279 (90.0)	340/368 (92.4)	251/268 (93.7)	591/636 (92.9)	0.86 (0.83-0.89)
LAVA-Flex	335/357 (93.8)	238/279 (85.3)	335/376 (89.1)	238/260 (91.5)	573/636 (90.1)	0.79 (0.75-0.83)

ldCT served as the reference standard. Denominators represent the total number regarding the respective statistical measure. PPV, positive predictive value; NPV, negative predictive value; ZTE, zero echo time; LAVA-Flex, Liver Acquisition with Volume Acceleration-flexible.

**Table 5:** Contingency table analysis comparing the positivity of detecting structural lesions between ZTE and LAVA-Flex.

	Degree	ZTE(+) LAVA (+)	ZTE(+) LAVA(-)	ZTE(-) LAVA(+)	ZTE(-) LAVA(-)	P value
Erosions	1	169	153	15	95	<0.001
	2	223	55	1	5	<0.001
	3	979	3	6	6	0.505
Sclerosis	1	440	176	44	20	<0.001
Joint space changes	1	216	2	4	11	0.683
	≧2	113	9	2	0	0.070

ZTE, zero echo time; LAVA-Flex, liver acquisition volume acceleration-flexible.

**Figure 4:**
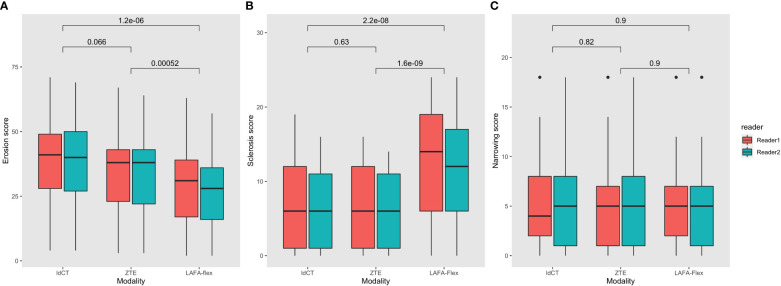
Comparison of sum scores of structural lesions of the sacroiliac joint with ldCT, ZTE and LAVA-Flex. **(A)** Erosions; **(B)** Sclerosis; **(C)** Joint space changes. ldCT, low-dose CT; ZTE, Zero echo time; LAVA-Flex, Liver Acquisition with Volume Acceleration-flexible.

The sum scores of different structural lesions with ldCT, ZTE and LAVA-Flex could be seen in **Table 6** and **Figure 4**, while comparisons of diagnostic confidence could be seen in **Table 6** and **Supplementary Materials 1**. The median sum scores of the three modalities were 40.5, 38 and 29, respectively. There is no significant difference between the sum score of erosions between ldCT and ZTE (p=0.066), but sum scores of both modalities were significantly higher than LAVA-Flex(p<0.001) The inter-reader κ values of ldCT and ZTE in detecting erosions were 0.87 and 0.81, indicating very good agreement between readers, while the inter-reader κ value of LAVA-Flex was 0.72, indicating good inter-reader agreement (**Table 6**).

**Table 6:** Sum score, diagnostic confidence and inter-reader κ value of ldCT, ZTE and LAVA in the detection of structural lesions of the sacroiliac joint.

	ldCT	ZTE	LAVA-Flex	P value
Sum score ^a^				
Erosions	40.5 (27.25-49.75)	38 (22.25-43)	29 (16.25-37.75)	<0.001
Sclerosis	6 (1-11)	6 (1-11)	13.5 (6-18)	<0.001
Joint space changes	4.5 (1-8)	5 (1-7.75)	5 (1.25-7)	0.973
Diagnostic confidence ^a^				
Erosions	9 (8-9)	8 (7-8)	7 (7-8)	<0.001
Sclerosis	9 (8-9)	8 (7-9)	5 (4-6)	<0.001
Joint space changes	8 (8-9)	8 (7-8)	7 (7-8)	<0.001
Inter-reader κ value ^b^				
Erosions	0.87 (0.85-0.89)	0.81 (0.79-0.83)	0.72 (0.69-0.75)	
Sclerosis	0.82 (0.78-0.86)	0.83 (0.79-0.86)	0.72 (0.68-0.76)	
Joint space changes	0.77 (0.71-0.83)	0.74 (0.68-0.80)	0.70 (0.64-0.77)	

ldCT, low-dose CT; ZTE, zero echo time; LAVA-Flex, Liver Acquisition with Volume Acceleration-flexible.

a Data in parentheses are interquartile range.

b Data in parentheses are 95% CIs.

### Diagnostic accuracy of sclerosis

The analysis revealed a higher sensitivity (616/680 [90.6%] vs 484/680 [71.2%]), specificity (1848/1864 [99.1%] vs 1073/1864 [57.6%]), accuracy (2464/2544 [96.9%] vs 1557/2544 [61.2%]) and κ value (0.92 vs 0.22) of ZTE in the detection of sclerosis, in comparison with LAVA-Flex (**Table 4**). The contingency table analysis further substantiated the significant difference in the positivity of detecting sclerosis between ZTE and LAVA-Flex(p<0.001) (**Table 5**). An example demonstrating the improved detection of sclerosis by ZTE is provided in **Figure 3**.

The median sum scores of sclerosis based on ldCT, ZTE and LAVA-Flex were 6, 6 and 13.5, respectively. The sum scores of sclerosis were not significantly different between ldCT and ZTE (p=0.63), but the sum score of LAVA-Flex is significantly higher than ldCT (p<0.001) and ZTE (p<0.001). The κ values of ldCT and ZTE were 0.82 and 0.83, both indicating very good agreement between the two readers. The κ value of LAVA-Flex was 0.72, suggesting good inter-reader agreement (**Table 6**).

### Diagnostic accuracy of joint space changes

Results of the analysis showed a comparable sensitivity (340/357 [95.2%] vs 335/357 [93.8%]), specificity (251/279 [90.0%] vs 238/279 [85.3%]), accuracy (591/636 [92.9%] vs 573/636 [90.1%]) and κ value (0.86 vs 0.79) between ZTE and LAVA-Flex in the detection of joint space changes (**Table 4**). Contingency table analysis failed to identify any significant difference between ZTE and LAVA-Flex (p=0.332), either in detecting first-degree joint space changes (p=0.683) or in detecting joint space changes assigned ≥ 2 points (p=0.070) (**Table 5**).

The median sum scores of ldCT, ZTE and LAVA-Flex were 4.5, 5 and 5, respectively. The sum scores of joint space changes were also comparable between each modality. (p=0.973) The κ values of ldCT, ZTE and LAVA-Flex were 0.77, 0.74 and 0.70, respectively (**Table 6**).

## Discussion

This study directly compared ZTE and T1w 3D-GRE sequences in the detection of structural lesions in the SIJ. Similar to the previous studies (7, 8), the assessment of the structural lesions was conducted using the scoring system developed by Diekhoff et al. (14) It should be noted that the scoring of sclerosis was modified in response to an observation during the training sessions that sclerosis with a depth less than 5 mm was very common in the upper sacral quadrant, which should not be considered pathological, since such findings are also present in healthy individuals. Based on this observation, the image evaluation panel revised the scoring criteria (**Table 2**). Another important distinction of this study was that all the analysis was conducted on the quadrant level (erosions and sclerosis) or SIJ on a single slice (joint space changes), as opposed to the patient level or joint level. By limiting the analysis on the quadrant level or SIJ level, the diagnostic accuracy could be exhibited more truthfully.

By using ldCT as the reference standard, we revealed that ZTE was more accurate than LAVA-Flex in the detection of erosions and sclerosis, but not joint space changes. More specifically, ZTE had an edge over LAVA-Flex in the visualization of low-degree erosions. This advantage could be attributed to the application of zero echo time, enabling the acquisition of sparse MR signal of cortical bone, since cortical bone tissue had a very short T2 relaxation time (6). ZTE has been acclaimed for its CT-like bone contrast, hence the gaining interest in this sequence in the field of musculoskeletal imaging (15). This study substantiated that ZTE could provide a precise depiction of the SIJ cortical bone contour. Despite the fact that the current definition of a positive SIJ MRI maintained indispensably the bone marrow edema or osteitis (4), this finding is still increasingly relevant given the mounting evidence that structural lesions, especially erosions, have incremental diagnostic value in the imaging assessment of axSpA (16, 17).

Previous studies by Diekhoff et al. and Baraliakos et al. reported that MR-VIBE had a sensitivity of 95% and 71.2% (7, 8), while our study reported a sensitivity of 81.5% in the detection of erosions. The study by Diekhoff et al. conducted the analysis of diagnostic performance on the patient level, which could lead to the overestimation of sensitivity. Similar to the study by Baraliakos et al., our study conducted analysis on the quadrant level or SIJ level, but it should be noted that the disease duration of our study is longer, hence the higher proportion of third-degree erosions. Consequently, our study reported a higher sensitivity (81.5%) and specificity (97.0%) of LAVA-Flex. In the meanwhile, Li et al. made an initial attempt to analyze the diagnostic performance of ZTE and reported its sensitivity to be 98.2%, but this sensitivity was calculated on the joint level (18). The accuracy (95.0%) and κ value (0.88) reported were similar to our study, which reported an accuracy of 94.0% and a κ value of 0.73.

The advantage of ZTE over LAVA-Flex in the detection of sclerosis was formidable. Our study reported a sensitivity of 90.6% and a specificity of 99.1% of ZTE in the detection of sclerosis, while the sensitivity and specificity for LAVA-Flex were only 71.2% and 57.6%. Neither of the two previous studies analyzed its capacity in detecting sclerosis (7, 8). T1w 3D-GRE is inherently not an appropriate sequence for the detection of sclerosis. Recognition of sclerosis on LAVA-Flex images depends on the finding of low signal, yet such low signal could easily be confused with fat deposition. In contrast, ZTE could render a near-CT visualization of sclerosis in the cortical bone. Alas, our study failed to prove the advantage of ZTE over LAVA-Flex in the detection of joint space changes.

There are certain limitations to our study. First, the sample size was relatively small with only 53 patients, with no healthy control for ethical consideration that CT could inflict ionizing radiation. Second, the MRI examinations were carried out on a 3.0-T MRI scanner; therefore, our result should not be easily extrapolated to other scanners with different magnetic field strength. Noteworthy, there are several novel MRI sequences with CT-like contrast. The study by Deppe et al. concluded that susceptibility-weighted imaging (SWI) depicts erosions and sclerosis more accurately than T1 spin echo MRI (19). Jans et al. developed the MRI-based synthetic CT and demonstrated its superiority to T1-weighted MRI in the detection of structural lesions in SIJ. It is desirable to comprehensively evaluate these 3D sequences in the detection of SIJ lesions (20).

In conclusion, ZTE could generate a near-CT visualization of the SIJ and improve the detection of low-degree erosions and sclerosis in the SIJ with excellent reliability in comparison to T1w 3D-GRE(LAVA). ZTE could be incorporated into the routine MRI examination of the SIJ for patients suspected of axSpA.

## Data availability statement

The raw data supporting the conclusions of this article will be made available by the authors, without undue reservation.

## Ethics statement

The studies involving human participants were reviewed and approved by the Ethical Committee of the Third Affiliated Hospital of Sun Yat-sen University. The patients/participants provided their written informed consent to participate in this study.

## Author contributions

Both CL and DL performed the imaging evaluation. CL devised the study design, while DL conducted the statistical analysis and drafted the manuscript. HW was in charge of the acquisition of the imaging data. BL and LT assisted in collecting patient information and helped coordinate this study. JG contributed conceptually to the project and was in charge of the integrity of this study. All authors contributed to the article and approved the submitted version.
