# Supplementary material for: Zero echo time MRI improved detection of erosions and sclerosis in the sacroiliac joint in comparison with LAVA-flex
Source: Front Endocrinol (Lausanne). 2023 May 29;14:1167334. doi: 10.3389/fendo.2023.1167334 (PMC10258343; doi:10.3389/fendo.2023.1167334)
Supplement: Supplementary file 1 [file Image_1.pdf]

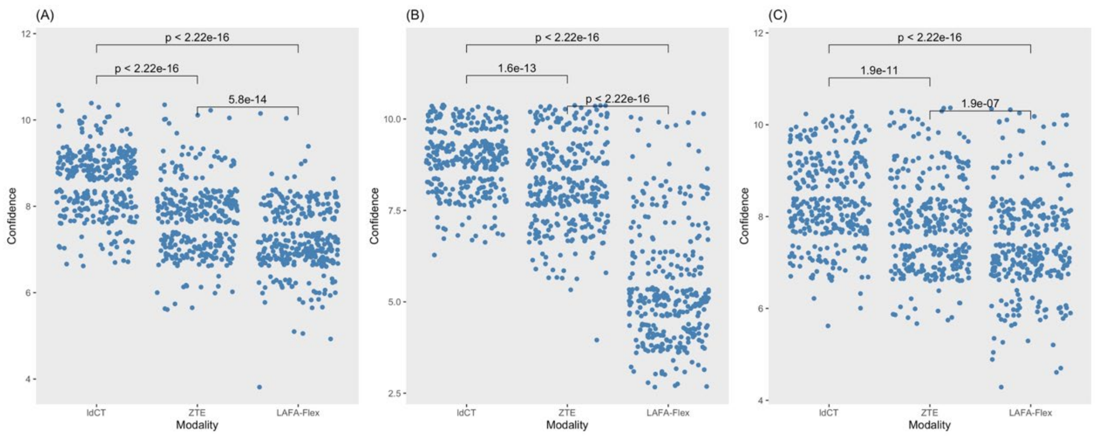

Supplementary materials Figure 1. Comparison of diagnostic confidence regarding the structural lesions of the sacroiliac joint with ldCT, ZTE and LAVA-Flex. (A) Erosions; (B) Sclerosis; (C) Joint space changes. ldCT = low-dose CT; ZTE = Zero echo time; LAVA-Flex = Liver Acquisition with Volume Acceleration-flexible.
